# 3,5-Diamino-4*H*-1,2,4-triazol-1-ium hydroxonium bis­(pyridine-2,6-di­carboxyl­ato)cobaltate(II) pyridine-2,6-dicarb­oxy­lic acid monohydrate

**DOI:** 10.1107/S1600536811027917

**Published:** 2011-07-16

**Authors:** S. Yousuf, A. S. Johnson, S. A. Kazmi, Madhukar Hemamalini, Hoong-Kun Fun

**Affiliations:** a H.E.J. Research Institute of Chemistry, International Center for Chemical and Biological Sciences, University of Karachi 75270, Pakistan; b Department of Pure and Applied Chemistry, University of Calabar, Calabar, PMB 1115, Nigeria; c X-ray Crystallography Unit, School of Physics, Universiti Sains Malaysia, 11800 USM, Penang, Malaysia

## Abstract

The asymmetric unit of the title complex, (C_2_H_6_N_5_)(H_3_O)[Co(C_7_H_3_NO_4_)_2_]·C_7_H_5_NO_4_·H_2_O, contains a Co^II^ ion coordin­ated by four O atoms and two N atoms from two dipicolinate ligands in a disorted octa­hedral environment, a protonated triazole mol­ecule, a neutral pyridine-2,6-dicarb­oxy­lic acid mol­ecule, a hydroxonium ion and a solvent water mol­ecule. In the crystal, the components are linked into a three-dimensional framework by inter­molecular O—H⋯O, N—H⋯O and N—H⋯N and weak C—H⋯O hydrogen bonds. In addition, π–π stacking inter­actions with centroid–centroid distances in the range 3.4809 (7)–3.8145 (6) Å are observed.

## Related literature

For the different coordination modes for transition metal–dipicolinate complexes, see: Quaglieri *et al.* (1972 ▶); Hakansson *et al.* (1993 ▶); Okabe & Oya (2000 ▶); Aghajani *et al.* (2009 ▶). For crystal structures of related complexes, see: Yousuf *et al.* (2011 ▶); Aghabozorg *et al.* (2009 ▶); Ramos Silva *et al.* (2008 ▶); Wang *et al.* (2004 ▶); MacDonald *et al.* (2000 ▶, 2004 ▶). For the stability of the temperature controller used in the data collection, see: Cosier & Glazer (1986 ▶).
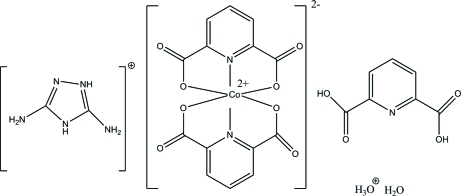


## Experimental

### 

#### Crystal data

(C_2_H_6_N_5_)(H_3_O)[Co(C_7_H_3_NO_4_)_2_]·C_7_H_5_NO_4_·H_2_O
*M* *_r_* = 693.42
Triclinic, 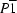

*a* = 8.0209 (2) Å
*b* = 9.2028 (2) Å
*c* = 18.7004 (4) Å
α = 98.536 (1)°
β = 96.721 (1)°
γ = 100.515 (1)°
*V* = 1327.32 (5) Å^3^
*Z* = 2
Mo *K*α radiation
μ = 0.74 mm^−1^
*T* = 100 K
0.78 × 0.59 × 0.35 mm

#### Data collection

Bruker SMART APEXII CCD area-detector diffractometer
Absorption correction: multi-scan (*SADABS*; Bruker, 2009 ▶) *T*_min_ = 0.595, *T*_max_ = 0.781
25547 measured reflections
7698 independent reflections
7360 reflections with *I* > 2σ(*I*)
*R*_int_ = 0.018

#### Refinement

*R*[*F* ^2^ > 2σ(*F* ^2^)] = 0.027
*wR*(*F* ^2^) = 0.072
*S* = 1.05
7698 reflections
467 parameters
H atoms treated by a mixture of independent and constrained refinement
Δρ_max_ = 0.48 e Å^−3^
Δρ_min_ = −0.43 e Å^−3^

### 

Data collection: *APEX2* (Bruker, 2009 ▶); cell refinement: *SAINT* (Bruker, 2009 ▶); data reduction: *SAINT*; program(s) used to solve structure: *SHELXS97* (Sheldrick, 2008 ▶); program(s) used to refine structure: *SHELXL97* (Sheldrick, 2008 ▶); molecular graphics: *SHELXTL* (Sheldrick, 2008 ▶) and *PLATON* (Spek, 2009 ▶); software used to prepare material for publication: *SHELXTL* and *PLATON*.

## Supplementary Material

Crystal structure: contains datablock(s) global, I. DOI: 10.1107/S1600536811027917/lh5282sup1.cif

Structure factors: contains datablock(s) I. DOI: 10.1107/S1600536811027917/lh5282Isup2.hkl

Additional supplementary materials:  crystallographic information; 3D view; checkCIF report

## Figures and Tables

**Table 1:** Hydrogen-bond geometry (Å, °)

*D*—H⋯*A*	*D*—H	H⋯*A*	*D*⋯*A*	*D*—H⋯*A*
N3—H1*N*3⋯O10^i^	0.841 (19)	2.249 (18)	3.0304 (15)	154.5 (17)
N3—H1*N*3⋯N8^i^	0.841 (19)	2.47 (2)	3.1379 (15)	136.5 (16)
N3—H2*N*3⋯O11^i^	0.86 (2)	2.28 (2)	2.9563 (15)	135.7 (17)
N4—H1*N*4⋯O2	0.85 (2)	2.072 (19)	2.9084 (14)	169 (2)
N4—H2*N*4⋯O1*W*	0.85 (2)	1.96 (2)	2.8057 (16)	172 (2)
N6—H1*N*6⋯O3^ii^	0.877 (18)	1.870 (18)	2.7368 (12)	169.7 (16)
N7—H1*N*7⋯O7	0.85 (2)	1.89 (2)	2.7245 (13)	167 (2)
O10—H1*OA*⋯O7^i^	0.79 (2)	1.86 (2)	2.5775 (13)	152 (3)
O1*W*—H1*W*1⋯O6^iii^	0.78 (2)	2.06 (2)	2.8315 (14)	172 (2)
O1*W*—H2*W*1⋯O1^ii^	0.82 (3)	2.23 (3)	2.8901 (14)	138 (2)
O2*W*—H2*W*2⋯O8^iv^	0.87 (3)	1.67 (2)	2.5279 (13)	170 (2)
O2*W*—H1*W*2⋯O5^v^	0.98 (3)	1.48 (3)	2.4622 (13)	177 (3)
O2*W*—H3*W*2⋯O6^ii^	0.87 (2)	1.71 (2)	2.5746 (13)	174 (2)
O12—H12*B*⋯N5^vi^	0.89 (2)	1.76 (2)	2.6303 (14)	169 (2)
C3—H3*A*⋯O9^vii^	0.95	2.52	3.1725 (14)	126
C5—H5*A*⋯O9^i^	0.95	2.59	3.5380 (15)	175
C10—H10*A*⋯O1^viii^	0.95	2.38	3.2669 (14)	156
C19—H19*A*⋯O2*W*	0.95	2.50	3.4269 (15)	165

Symmetry codes: (i) 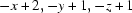; (ii) 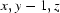; (iii) 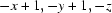; (iv) 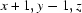; (v) 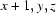; (vi) 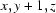; (vii) 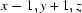; (viii) 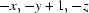.

**Fig. 1.**
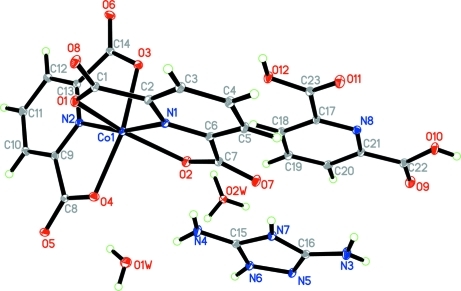
The asymmetric unit of (I) showing 30% probability displacement ellipsoids.

**Fig. 2.**
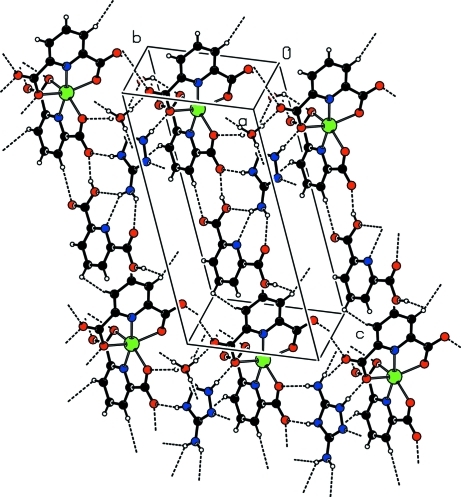
The crystal packing of the title compound, showing hydrogen bonds as dashed lines.

## supplementary crystallographic information

### Comment

Pyridine-2,6 dicarboxylic acid (H2dipic) and its anions (dipic^2-^ & dipicH^-^) have been identified to be well suited for the construction of multidimensional frameworks with simple metal ions and non-metal cations due to the presence of heterocyclic nitrogen atoms and two carboxylate groups. A range of different coordination modes exists in transition metal-dipicolinate complexes (Quaglieri *et al.*, 1972, Hakansson *et al.*, 1993, Okabe & Oya, 2000) depending on whether the anionic or protonated forms of the carboxylates are coordinated to the metal ion. It has been observed that providing suitable conditions for the transfer of acidic protons to appropriate bases will result in increased intermolecular interactions and stabilization of the resulting system (Aghajani *et al.*, 2009). In recent work on the study of the structural features of molecules containing metal chelate and triazole rings, we have determined the crystal structure of a Cu(II) with a derivative of 1,2,4-triazole and dipicolinic acid (Yousuf *et al.*, 2011). Herein, we report the crystal structure of the title complex (I).

The asymmetric unit of (I) is shown in Fig. 1. It contains a Co^II^ ion, two tridentate dipicolinte ligands, a protonated triazole molecule, a neutral pyridine 2,6-dicarboxylic acid molecule, a hydroxonium ion and a solvent water molecule. The Co^II^ ion is in a distorted octahedral coordination environment formed by four oxygen atoms and two nitrogen atoms from two dipicolinate ligands. The two dipiconilate ligands are assembled approximately perpendicular to each other around the Co^II^ ion with atoms O3 and O4 in the axial sites and atoms N1/N2/O1/O2 forming the equatorial sites. All bond lengths are in agreement with those common to related structures (Yousuf *et al.*, 2011; Aghabozorg *et al.*, 2009; Ramos Silva *et al.*, 2008; Wang *et al.*, 2004; MacDonald *et al.*, 2000,2004).

In the crystal structure (Fig. 2), intermolecular O—H···O, N—H···O, N—H···N and weak C—H···O (Table 1) hydrogen bonds form a three-dimensional network. The network is further stabilized by π–π stacking interactions between the centroids of Co1/O2/N1/C6/C7 (*Cg*1), Co1/O3/N2/C13/C14 (*Cg*2), N1/C2–C6 (*Cg*3), N2/C9–C13 (*Cg*4), N8/C17–C21 (*Cg*5) and N5–N7/C15/C16 (*Cg*6) rings, with *Cg*1···*Cg*5^i^, *Cg*2···*Cg*4^ii^, *Cg*3···*Cg*5^iii^, *Cg*3···*Cg*6^iv^ and *Cg*4···*Cg*4^v^, distances of 3.6235 (6) Å, 3.8145 (6) Å, 3.7500 (7) Å, 3.5247 (7) Å and 3.4809 (7) Å, respectively [symmetry codes: (i) -1+x, y, z; (ii) 1-x, 1-y, -z; (iii) -1+x, y, z; (iv) x, -1+y, z; (v) -x, 1-y, -z].

### Experimental

Pyridine-2,6-dicarboxylic acid and 3,5-diamino-1,2,4-triazole were purchased from Merck and Molekula respectively. Cobaltchloride hexahydrate (CoCl_2_.6H_2_O) and HPLC grade methanol were Uni-Chem and *M* TEDIA products, respectively. Deionized water was also used in the procedures when needed.

A method similar to that reported by Yousuf *et al.* (2011) was used. 1 mmol (0.099 g) of 3,5-diamino- 1,2,4-triazole and 1 mmol of dipicolinic acid (0.167 g) were dissolved in a mixture of methanol/water solution (1:10, 11 ml). The resulting solution was heated to 338 K with stirring. An aqueous solution (1 ml) containing 0.5 mmol (0.119 g) of CoCl_2_.6H_2_O was added to the stirred solution. The redish purple suspension was allowed to stir further for 1 hr, and then filtered while hot. The filtrate was kept at room temperature. Well shaped purple crystals of the title compound were formed by slow evaporation of the solution after 2 weeks. Percentage yield based on cobalt is 46.34%.

### Refinement

The N– and O-bound H atoms were located from a difference map and refined freely [N–H = 0.841 (19)–0.877 (18) Å and O–H = 0.78 (2)–0.98 (3) Å]. The remaining H atoms were positioned geometrically [C–H = 0.95 Å] and were refined using a riding model, with *U*_iso_(H) = 1.2*U*_eq_(C). One reflection (0 0 1) blocked by the beamstop and two outlier reflections (-2 -5 24) and (2 - 5 24) were omitted.

### Crystal data

**Table:**

(C_2_H_6_N_5_)(H_3_O)[Co(C_7_H_3_NO_4_)_2_]·C_7_H_5_NO_4_·H_2_O	*Z* = 2
*M**_r_* = 693.42	*F*(000) = 710
Triclinic, *P*1	*D*_x_ = 1.735 Mg m^−^^3^
Hall symbol: -P 1	Mo *K*α radiation, λ = 0.71073 Å
*a* = 8.0209 (2) Å	Cell parameters from 9943 reflections
*b* = 9.2028 (2) Å	θ = 2.9–35.1°
*c* = 18.7004 (4) Å	µ = 0.74 mm^−^^1^
α = 98.536 (1)°	*T* = 100 K
β = 96.721 (1)°	Block, purple
γ = 100.515 (1)°	0.78 × 0.59 × 0.35 mm
*V* = 1327.32 (5) Å^3^	

### Data collection

**Table:**

Bruker SMART APEXII CCD area-detector diffractometer	7698 independent reflections
Radiation source: fine-focus sealed tube	7360 reflections with *I* > 2σ(*I*)
graphite	*R*_int_ = 0.018
φ and ω scans	θ_max_ = 30.0°, θ_min_ = 2.2°
Absorption correction: multi-scan (*SADABS*; Bruker, 2009)	*h* = −11→11
*T*_min_ = 0.595, *T*_max_ = 0.781	*k* = −12→12
25547 measured reflections	*l* = −26→26

### Refinement

**Table:**

Refinement on *F*^2^	Primary atom site location: structure-invariant direct methods
Least-squares matrix: full	Secondary atom site location: difference Fourier map
*R*[*F*^2^ > 2σ(*F*^2^)] = 0.027	Hydrogen site location: inferred from neighbouring sites
*wR*(*F*^2^) = 0.072	H atoms treated by a mixture of independent and constrained refinement
*S* = 1.05	*w* = 1/[σ^2^(*F*_o_^2^) + (0.0325*P*)^2^ + 0.8694*P*] where *P* = (*F*_o_^2^ + 2*F*_c_^2^)/3
7698 reflections	(Δ/σ)_max_ = 0.001
467 parameters	Δρ_max_ = 0.48 e Å^−^^3^
0 restraints	Δρ_min_ = −0.43 e Å^−^^3^

### Special details

**Table:**

Experimental. The crystal was placed in the cold stream of an Oxford Cryosystems Cobra open-flow nitrogen cryostat (Cosier & Glazer, 1986) operating at 100.0 (1) K.
Geometry. All s.u.'s (except the s.u. in the dihedral angle between two l.s. planes) are estimated using the full covariance matrix. The cell s.u.'s are taken into account individually in the estimation of s.u.'s in distances, angles and torsion angles; correlations between s.u.'s in cell parameters are only used when they are defined by crystal symmetry. An approximate (isotropic) treatment of cell s.u.'s is used for estimating s.u.'s involving l.s. planes.
Refinement. Refinement of *F*^2^ against ALL reflections. The weighted *R*-factor *wR* and goodness of fit *S* are based on *F*^2^, conventional *R*-factors *R* are based on *F*, with *F* set to zero for negative *F*^2^. The threshold expression of *F*^2^ > 2σ(*F*^2^) is used only for calculating *R*-factors(gt) *etc*. and is not relevant to the choice of reflections for refinement. *R*-factors based on *F*^2^ are statistically about twice as large as those based on *F*, and *R*- factors based on ALL data will be even larger.

### Fractional atomic coordinates and isotropic or equivalent isotropic displacement parameters (Å^2^)

**Table:**

	*x*	*y*	*z*	*U*_iso_*/*U*_eq_	
Co1	0.302228 (19)	0.644423 (16)	0.169857 (8)	0.01036 (5)	
N1	0.31342 (12)	0.75361 (10)	0.27349 (5)	0.01027 (16)	
N2	0.30641 (12)	0.58813 (10)	0.06050 (5)	0.01004 (16)	
O1	0.13428 (11)	0.80434 (9)	0.15754 (4)	0.01381 (15)	
O2	0.45499 (11)	0.52726 (9)	0.23827 (4)	0.01373 (15)	
O3	0.52409 (11)	0.80151 (9)	0.14717 (4)	0.01281 (15)	
O4	0.12604 (11)	0.43393 (9)	0.13837 (5)	0.01510 (16)	
O5	0.00050 (11)	0.25528 (9)	0.04207 (5)	0.01481 (16)	
O6	0.63852 (12)	0.88973 (10)	0.05403 (5)	0.01749 (17)	
O7	0.54563 (11)	0.51915 (9)	0.35540 (5)	0.01517 (16)	
O8	0.06823 (12)	1.00920 (10)	0.22102 (5)	0.01651 (16)	
C1	0.13704 (14)	0.89743 (12)	0.21521 (6)	0.01192 (19)	
C2	0.23804 (14)	0.87158 (12)	0.28416 (6)	0.01092 (18)	
C3	0.25593 (15)	0.95939 (12)	0.35273 (6)	0.01309 (19)	
H3A	0.2025	1.0434	0.3598	0.016*	
C4	0.35408 (15)	0.92139 (13)	0.41088 (6)	0.0138 (2)	
H4A	0.3688	0.9801	0.4583	0.017*	
C5	0.43087 (15)	0.79723 (12)	0.39962 (6)	0.01307 (19)	
H5A	0.4972	0.7692	0.4388	0.016*	
C6	0.40713 (14)	0.71590 (12)	0.32909 (6)	0.01085 (18)	
C7	0.47660 (14)	0.57714 (12)	0.30615 (6)	0.01145 (19)	
C8	0.09977 (14)	0.37796 (12)	0.07162 (6)	0.01156 (19)	
C9	0.19911 (13)	0.46510 (12)	0.02260 (6)	0.01047 (18)	
C10	0.19089 (14)	0.42474 (12)	−0.05245 (6)	0.01231 (19)	
H10A	0.1149	0.3366	−0.0788	0.015*	
C11	0.29789 (15)	0.51793 (13)	−0.08794 (6)	0.0145 (2)	
H11A	0.2944	0.4944	−0.1394	0.017*	
C12	0.41009 (15)	0.64579 (13)	−0.04790 (6)	0.0135 (2)	
H12A	0.4835	0.7103	−0.0714	0.016*	
C13	0.41151 (14)	0.67618 (12)	0.02725 (6)	0.01103 (18)	
C14	0.53451 (14)	0.80151 (12)	0.07972 (6)	0.01191 (19)	
O9	1.31990 (13)	0.28490 (10)	0.44827 (5)	0.01975 (18)	
O10	1.29486 (12)	0.49673 (10)	0.51898 (5)	0.01785 (17)	
O11	0.93965 (12)	0.82190 (10)	0.44184 (5)	0.01879 (17)	
O12	0.83933 (12)	0.78120 (10)	0.32170 (5)	0.01702 (17)	
N8	1.08308 (12)	0.56516 (10)	0.41642 (5)	0.01197 (17)	
C17	0.97897 (14)	0.60148 (12)	0.36347 (6)	0.01172 (19)	
C18	0.92791 (15)	0.51390 (13)	0.29409 (6)	0.0144 (2)	
H18A	0.8533	0.5439	0.2583	0.017*	
C19	0.98785 (16)	0.38218 (13)	0.27808 (6)	0.0150 (2)	
H19A	0.9538	0.3196	0.2315	0.018*	
C20	1.09842 (15)	0.34425 (12)	0.33159 (6)	0.0144 (2)	
H20A	1.1440	0.2562	0.3223	0.017*	
C21	1.14112 (14)	0.43846 (12)	0.39946 (6)	0.01212 (19)	
C22	1.26029 (15)	0.39687 (13)	0.45743 (6)	0.0139 (2)	
C23	0.91764 (14)	0.74663 (12)	0.38133 (6)	0.01276 (19)	
N3	0.79103 (15)	0.19736 (13)	0.43972 (6)	0.0187 (2)	
N4	0.47746 (15)	0.21239 (12)	0.21219 (6)	0.0187 (2)	
N5	0.72357 (13)	0.03157 (11)	0.32639 (5)	0.01330 (18)	
N6	0.62946 (13)	0.03978 (11)	0.25973 (5)	0.01312 (17)	
N7	0.62630 (13)	0.24252 (11)	0.33308 (5)	0.01265 (17)	
C15	0.57107 (15)	0.16592 (12)	0.26417 (6)	0.01257 (19)	
C16	0.71983 (14)	0.15723 (12)	0.36943 (6)	0.01230 (19)	
O1W	0.29638 (15)	0.03156 (12)	0.08229 (6)	0.0269 (2)	
O2W	0.87798 (12)	0.10223 (10)	0.12792 (5)	0.01508 (16)	
H1N3	0.795 (2)	0.285 (2)	0.4615 (11)	0.027 (5)*	
H2N3	0.863 (3)	0.147 (2)	0.4562 (11)	0.036 (5)*	
H1N4	0.457 (3)	0.300 (2)	0.2214 (11)	0.033 (5)*	
H2N4	0.430 (3)	0.152 (2)	0.1733 (11)	0.027 (5)*	
H1N6	0.609 (2)	−0.036 (2)	0.2233 (10)	0.024 (4)*	
H1N7	0.605 (3)	0.328 (2)	0.3472 (11)	0.031 (5)*	
H1OA	1.351 (3)	0.468 (3)	0.5490 (12)	0.041 (6)*	
H12B	0.806 (3)	0.868 (2)	0.3295 (11)	0.036 (5)*	
H1W1	0.306 (3)	0.047 (3)	0.0429 (13)	0.043 (6)*	
H2W1	0.218 (3)	−0.039 (3)	0.0821 (12)	0.039 (6)*	
H2W2	0.947 (3)	0.065 (3)	0.1557 (13)	0.044 (6)*	
H1W2	0.930 (4)	0.165 (3)	0.0952 (16)	0.079 (9)*	
H3W2	0.796 (3)	0.028 (2)	0.1059 (12)	0.036 (5)*	

### Atomic displacement parameters (Å^2^)

**Table:**

	*U*^11^	*U*^22^	*U*^33^	*U*^12^	*U*^13^	*U*^23^
Co1	0.01302 (7)	0.00984 (7)	0.00789 (7)	0.00244 (5)	0.00097 (5)	0.00086 (5)
N1	0.0119 (4)	0.0096 (4)	0.0093 (4)	0.0025 (3)	0.0011 (3)	0.0019 (3)
N2	0.0109 (4)	0.0096 (4)	0.0093 (4)	0.0019 (3)	0.0007 (3)	0.0013 (3)
O1	0.0170 (4)	0.0134 (4)	0.0104 (4)	0.0044 (3)	−0.0007 (3)	0.0008 (3)
O2	0.0183 (4)	0.0124 (4)	0.0110 (4)	0.0059 (3)	0.0014 (3)	0.0010 (3)
O3	0.0158 (4)	0.0112 (3)	0.0099 (3)	0.0005 (3)	0.0005 (3)	0.0007 (3)
O4	0.0187 (4)	0.0136 (4)	0.0113 (4)	−0.0010 (3)	0.0022 (3)	0.0020 (3)
O5	0.0165 (4)	0.0115 (4)	0.0142 (4)	−0.0016 (3)	0.0001 (3)	0.0023 (3)
O6	0.0186 (4)	0.0165 (4)	0.0138 (4)	−0.0050 (3)	0.0013 (3)	0.0030 (3)
O7	0.0203 (4)	0.0137 (4)	0.0125 (4)	0.0080 (3)	−0.0011 (3)	0.0025 (3)
O8	0.0218 (4)	0.0152 (4)	0.0144 (4)	0.0098 (3)	−0.0002 (3)	0.0033 (3)
C1	0.0130 (5)	0.0119 (5)	0.0110 (5)	0.0025 (4)	0.0006 (4)	0.0034 (4)
C2	0.0126 (4)	0.0104 (4)	0.0101 (4)	0.0030 (4)	0.0013 (4)	0.0026 (4)
C3	0.0168 (5)	0.0111 (4)	0.0118 (5)	0.0048 (4)	0.0020 (4)	0.0011 (4)
C4	0.0180 (5)	0.0126 (5)	0.0104 (5)	0.0038 (4)	0.0013 (4)	0.0003 (4)
C5	0.0160 (5)	0.0130 (5)	0.0103 (5)	0.0043 (4)	0.0000 (4)	0.0023 (4)
C6	0.0129 (4)	0.0096 (4)	0.0104 (4)	0.0027 (4)	0.0016 (4)	0.0024 (3)
C7	0.0123 (4)	0.0104 (4)	0.0118 (5)	0.0031 (4)	0.0010 (4)	0.0022 (4)
C8	0.0114 (4)	0.0111 (4)	0.0125 (5)	0.0026 (4)	0.0007 (4)	0.0034 (4)
C9	0.0098 (4)	0.0104 (4)	0.0111 (5)	0.0025 (3)	0.0009 (3)	0.0016 (3)
C10	0.0120 (5)	0.0124 (5)	0.0112 (5)	0.0020 (4)	−0.0002 (4)	−0.0001 (4)
C11	0.0156 (5)	0.0172 (5)	0.0097 (5)	0.0024 (4)	0.0013 (4)	0.0011 (4)
C12	0.0143 (5)	0.0151 (5)	0.0108 (5)	0.0012 (4)	0.0019 (4)	0.0029 (4)
C13	0.0110 (4)	0.0104 (4)	0.0110 (5)	0.0014 (4)	0.0008 (4)	0.0013 (3)
C14	0.0128 (5)	0.0106 (4)	0.0112 (5)	0.0014 (4)	0.0004 (4)	0.0009 (4)
O9	0.0265 (5)	0.0157 (4)	0.0186 (4)	0.0114 (3)	0.0001 (3)	0.0019 (3)
O10	0.0241 (4)	0.0166 (4)	0.0129 (4)	0.0101 (3)	−0.0040 (3)	0.0006 (3)
O11	0.0241 (4)	0.0176 (4)	0.0149 (4)	0.0096 (3)	−0.0003 (3)	−0.0005 (3)
O12	0.0231 (4)	0.0135 (4)	0.0149 (4)	0.0088 (3)	−0.0028 (3)	0.0020 (3)
N8	0.0135 (4)	0.0112 (4)	0.0117 (4)	0.0038 (3)	0.0014 (3)	0.0022 (3)
C17	0.0130 (5)	0.0109 (4)	0.0114 (5)	0.0032 (4)	0.0013 (4)	0.0021 (4)
C18	0.0172 (5)	0.0141 (5)	0.0112 (5)	0.0036 (4)	−0.0005 (4)	0.0019 (4)
C19	0.0201 (5)	0.0134 (5)	0.0111 (5)	0.0031 (4)	0.0024 (4)	0.0008 (4)
C20	0.0185 (5)	0.0112 (5)	0.0142 (5)	0.0044 (4)	0.0036 (4)	0.0021 (4)
C21	0.0139 (5)	0.0116 (5)	0.0115 (5)	0.0036 (4)	0.0014 (4)	0.0033 (4)
C22	0.0159 (5)	0.0129 (5)	0.0131 (5)	0.0039 (4)	0.0012 (4)	0.0027 (4)
C23	0.0125 (5)	0.0125 (5)	0.0135 (5)	0.0036 (4)	0.0004 (4)	0.0029 (4)
N3	0.0260 (5)	0.0177 (5)	0.0116 (4)	0.0078 (4)	−0.0019 (4)	−0.0004 (4)
N4	0.0256 (5)	0.0145 (5)	0.0148 (5)	0.0072 (4)	−0.0046 (4)	0.0009 (4)
N5	0.0177 (4)	0.0120 (4)	0.0106 (4)	0.0050 (3)	0.0003 (3)	0.0021 (3)
N6	0.0180 (4)	0.0102 (4)	0.0105 (4)	0.0037 (3)	−0.0004 (3)	0.0006 (3)
N7	0.0170 (4)	0.0101 (4)	0.0109 (4)	0.0047 (3)	0.0008 (3)	0.0004 (3)
C15	0.0152 (5)	0.0099 (4)	0.0120 (5)	0.0017 (4)	0.0019 (4)	0.0013 (4)
C16	0.0145 (5)	0.0111 (4)	0.0116 (5)	0.0029 (4)	0.0022 (4)	0.0024 (4)
O1W	0.0335 (6)	0.0282 (5)	0.0137 (4)	−0.0070 (4)	−0.0010 (4)	0.0062 (4)
O2W	0.0156 (4)	0.0137 (4)	0.0141 (4)	−0.0003 (3)	−0.0010 (3)	0.0031 (3)

### Geometric parameters (Å, °)

**Table:**

Co1—N1	2.0302 (9)	O9—C22	1.2110 (14)
Co1—N2	2.0395 (9)	O10—C22	1.3283 (14)
Co1—O4	2.1363 (8)	O10—H1OA	0.78 (2)
Co1—O1	2.1875 (8)	O11—C23	1.2113 (14)
Co1—O2	2.1967 (8)	O12—C23	1.3242 (13)
Co1—O3	2.2037 (8)	O12—H12B	0.89 (2)
N1—C6	1.3363 (14)	N8—C17	1.3402 (14)
N1—C2	1.3371 (13)	N8—C21	1.3415 (14)
N2—C13	1.3329 (14)	C17—C18	1.3949 (15)
N2—C9	1.3373 (13)	C17—C23	1.5112 (15)
O1—C1	1.2691 (13)	C18—C19	1.3888 (16)
O2—C7	1.2644 (13)	C18—H18A	0.9500
O3—C14	1.2738 (13)	C19—C20	1.3851 (16)
O4—C8	1.2553 (14)	C19—H19A	0.9500
O5—C8	1.2640 (13)	C20—C21	1.3939 (15)
O6—C14	1.2443 (14)	C20—H20A	0.9500
O7—C7	1.2546 (13)	C21—C22	1.4999 (15)
O8—C1	1.2500 (13)	N3—C16	1.3393 (15)
C1—C2	1.5141 (15)	N3—H1N3	0.84 (2)
C2—C3	1.3873 (15)	N3—H2N3	0.86 (2)
C3—C4	1.3920 (15)	N4—C15	1.3271 (15)
C3—H3A	0.9500	N4—H1N4	0.84 (2)
C4—C5	1.3946 (15)	N4—H2N4	0.85 (2)
C4—H4A	0.9500	N5—C16	1.3146 (14)
C5—C6	1.3901 (15)	N5—N6	1.3992 (13)
C5—H5A	0.9500	N6—C15	1.3238 (14)
C6—C7	1.5094 (15)	N6—H1N6	0.876 (19)
C8—C9	1.5081 (15)	N7—C15	1.3553 (14)
C9—C10	1.3882 (15)	N7—C16	1.3765 (14)
C10—C11	1.3950 (16)	N7—H1N7	0.84 (2)
C10—H10A	0.9500	O1W—H1W1	0.78 (2)
C11—C12	1.3962 (15)	O1W—H2W1	0.82 (2)
C11—H11A	0.9500	O2W—H2W2	0.86 (2)
C12—C13	1.3899 (15)	O2W—H1W2	0.98 (3)
C12—H12A	0.9500	O2W—H3W2	0.87 (2)
C13—C14	1.5128 (15)		
			
N1—Co1—N2	165.60 (4)	C12—C11—H11A	120.0
N1—Co1—O4	116.74 (4)	C13—C12—C11	118.22 (10)
N2—Co1—O4	75.79 (3)	C13—C12—H12A	120.9
N1—Co1—O1	76.28 (3)	C11—C12—H12A	120.9
N2—Co1—O1	94.70 (3)	N2—C13—C12	121.33 (10)
O4—Co1—O1	102.06 (3)	N2—C13—C14	113.26 (9)
N1—Co1—O2	75.36 (3)	C12—C13—C14	125.30 (10)
N2—Co1—O2	114.18 (3)	O6—C14—O3	126.51 (10)
O4—Co1—O2	85.99 (3)	O6—C14—C13	118.22 (10)
O1—Co1—O2	151.12 (3)	O3—C14—C13	115.21 (9)
N1—Co1—O3	93.99 (3)	C22—O10—H1OA	110.1 (16)
N2—Co1—O3	74.89 (3)	C23—O12—H12B	113.6 (13)
O4—Co1—O3	148.33 (3)	C17—N8—C21	116.54 (10)
O1—Co1—O3	92.04 (3)	N8—C17—C18	123.48 (10)
O2—Co1—O3	95.24 (3)	N8—C17—C23	116.87 (9)
C6—N1—C2	120.70 (9)	C18—C17—C23	119.64 (10)
C6—N1—Co1	120.21 (7)	C19—C18—C17	118.96 (10)
C2—N1—Co1	118.84 (7)	C19—C18—H18A	120.5
C13—N2—C9	120.81 (9)	C17—C18—H18A	120.5
C13—N2—Co1	119.95 (7)	C20—C19—C18	118.44 (10)
C9—N2—Co1	119.22 (7)	C20—C19—H19A	120.8
C1—O1—Co1	114.51 (7)	C18—C19—H19A	120.8
C7—O2—Co1	115.16 (7)	C19—C20—C21	118.38 (10)
C14—O3—Co1	115.29 (7)	C19—C20—H20A	120.8
C8—O4—Co1	116.54 (7)	C21—C20—H20A	120.8
O8—C1—O1	127.20 (10)	N8—C21—C20	124.19 (10)
O8—C1—C2	116.62 (10)	N8—C21—C22	117.51 (10)
O1—C1—C2	116.17 (9)	C20—C21—C22	118.30 (10)
N1—C2—C3	121.32 (10)	O9—C22—O10	123.98 (11)
N1—C2—C1	113.46 (9)	O9—C22—C21	123.16 (11)
C3—C2—C1	125.22 (10)	O10—C22—C21	112.85 (10)
C2—C3—C4	118.37 (10)	O11—C23—O12	124.81 (10)
C2—C3—H3A	120.8	O11—C23—C17	124.43 (10)
C4—C3—H3A	120.8	O12—C23—C17	110.74 (9)
C3—C4—C5	120.10 (10)	C16—N3—H1N3	119.3 (13)
C3—C4—H4A	120.0	C16—N3—H2N3	117.9 (14)
C5—C4—H4A	120.0	H1N3—N3—H2N3	118.9 (19)
C6—C5—C4	117.77 (10)	C15—N4—H1N4	117.7 (14)
C6—C5—H5A	121.1	C15—N4—H2N4	120.0 (13)
C4—C5—H5A	121.1	H1N4—N4—H2N4	121.8 (19)
N1—C6—C5	121.75 (10)	C16—N5—N6	104.54 (9)
N1—C6—C7	112.49 (9)	C15—N6—N5	110.78 (9)
C5—C6—C7	125.75 (10)	C15—N6—H1N6	128.7 (12)
O7—C7—O2	125.78 (10)	N5—N6—H1N6	120.4 (12)
O7—C7—C6	117.92 (10)	C15—N7—C16	107.23 (9)
O2—C7—C6	116.27 (9)	C15—N7—H1N7	122.3 (13)
O4—C8—O5	126.31 (10)	C16—N7—H1N7	130.4 (13)
O4—C8—C9	116.39 (9)	N6—C15—N4	127.91 (11)
O5—C8—C9	117.30 (10)	N6—C15—N7	106.84 (10)
N2—C9—C10	121.80 (10)	N4—C15—N7	125.25 (10)
N2—C9—C8	111.81 (9)	N5—C16—N3	125.83 (11)
C10—C9—C8	126.33 (10)	N5—C16—N7	110.61 (10)
C9—C10—C11	117.79 (10)	N3—C16—N7	123.53 (10)
C9—C10—H10A	121.1	H1W1—O1W—H2W1	112 (2)
C11—C10—H10A	121.1	H2W2—O2W—H1W2	117 (2)
C10—C11—C12	120.03 (10)	H2W2—O2W—H3W2	107 (2)
C10—C11—H11A	120.0	H1W2—O2W—H3W2	114 (2)
			
N2—Co1—N1—C6	−129.11 (14)	Co1—O2—C7—O7	173.12 (9)
O4—Co1—N1—C6	81.84 (9)	Co1—O2—C7—C6	−4.72 (12)
O1—Co1—N1—C6	178.61 (9)	N1—C6—C7—O7	−170.09 (10)
O2—Co1—N1—C6	4.12 (8)	C5—C6—C7—O7	8.47 (17)
O3—Co1—N1—C6	−90.27 (8)	N1—C6—C7—O2	7.92 (14)
N2—Co1—N1—C2	45.17 (19)	C5—C6—C7—O2	−173.51 (11)
O4—Co1—N1—C2	−103.88 (8)	Co1—O4—C8—O5	−179.79 (9)
O1—Co1—N1—C2	−7.12 (8)	Co1—O4—C8—C9	1.30 (12)
O2—Co1—N1—C2	178.39 (9)	C13—N2—C9—C10	−0.75 (16)
O3—Co1—N1—C2	84.01 (8)	Co1—N2—C9—C10	177.15 (8)
N1—Co1—N2—C13	30.87 (19)	C13—N2—C9—C8	176.77 (9)
O4—Co1—N2—C13	−177.41 (9)	Co1—N2—C9—C8	−5.33 (12)
O1—Co1—N2—C13	81.32 (8)	O4—C8—C9—N2	2.47 (14)
O2—Co1—N2—C13	−98.53 (8)	O5—C8—C9—N2	−176.54 (10)
O3—Co1—N2—C13	−9.53 (8)	O4—C8—C9—C10	179.86 (10)
N1—Co1—N2—C9	−147.05 (13)	O5—C8—C9—C10	0.85 (16)
O4—Co1—N2—C9	4.67 (8)	N2—C9—C10—C11	−0.58 (16)
O1—Co1—N2—C9	−96.60 (8)	C8—C9—C10—C11	−177.72 (10)
O2—Co1—N2—C9	83.55 (8)	C9—C10—C11—C12	0.86 (16)
O3—Co1—N2—C9	172.55 (9)	C10—C11—C12—C13	0.11 (17)
N1—Co1—O1—C1	7.60 (8)	C9—N2—C13—C12	1.79 (16)
N2—Co1—O1—C1	−161.02 (8)	Co1—N2—C13—C12	−176.09 (8)
O4—Co1—O1—C1	122.53 (8)	C9—N2—C13—C14	−174.64 (9)
O2—Co1—O1—C1	18.69 (12)	Co1—N2—C13—C14	7.48 (12)
O3—Co1—O1—C1	−86.02 (8)	C11—C12—C13—N2	−1.46 (17)
N1—Co1—O2—C7	0.71 (8)	C11—C12—C13—C14	174.52 (10)
N2—Co1—O2—C7	169.25 (8)	Co1—O3—C14—O6	172.81 (10)
O4—Co1—O2—C7	−118.28 (8)	Co1—O3—C14—C13	−10.06 (12)
O1—Co1—O2—C7	−10.43 (12)	N2—C13—C14—O6	179.76 (10)
O3—Co1—O2—C7	93.47 (8)	C12—C13—C14—O6	3.49 (17)
N1—Co1—O3—C14	−160.07 (8)	N2—C13—C14—O3	2.37 (14)
N2—Co1—O3—C14	10.63 (8)	C12—C13—C14—O3	−173.89 (11)
O4—Co1—O3—C14	33.44 (11)	C21—N8—C17—C18	1.17 (16)
O1—Co1—O3—C14	−83.69 (8)	C21—N8—C17—C23	−178.06 (10)
O2—Co1—O3—C14	124.29 (8)	N8—C17—C18—C19	−0.37 (18)
N1—Co1—O4—C8	169.32 (8)	C23—C17—C18—C19	178.84 (10)
N2—Co1—O4—C8	−3.10 (8)	C17—C18—C19—C20	−0.96 (17)
O1—Co1—O4—C8	88.76 (8)	C18—C19—C20—C21	1.41 (17)
O2—Co1—O4—C8	−119.28 (8)	C17—N8—C21—C20	−0.67 (17)
O3—Co1—O4—C8	−25.80 (12)	C17—N8—C21—C22	178.97 (10)
Co1—O1—C1—O8	171.75 (10)	C19—C20—C21—N8	−0.61 (18)
Co1—O1—C1—C2	−6.90 (12)	C19—C20—C21—C22	179.75 (10)
C6—N1—C2—C3	0.73 (16)	N8—C21—C22—O9	179.71 (11)
Co1—N1—C2—C3	−173.52 (8)	C20—C21—C22—O9	−0.62 (18)
C6—N1—C2—C1	−179.91 (9)	N8—C21—C22—O10	−1.23 (15)
Co1—N1—C2—C1	5.85 (12)	C20—C21—C22—O10	178.43 (10)
O8—C1—C2—N1	−177.62 (10)	N8—C17—C23—O11	−8.97 (17)
O1—C1—C2—N1	1.18 (14)	C18—C17—C23—O11	171.78 (12)
O8—C1—C2—C3	1.72 (17)	N8—C17—C23—O12	170.01 (10)
O1—C1—C2—C3	−179.48 (11)	C18—C17—C23—O12	−9.25 (15)
N1—C2—C3—C4	−0.38 (17)	C16—N5—N6—C15	−0.68 (13)
C1—C2—C3—C4	−179.67 (10)	N5—N6—C15—N4	179.92 (12)
C2—C3—C4—C5	−0.34 (17)	N5—N6—C15—N7	0.39 (13)
C3—C4—C5—C6	0.70 (17)	C16—N7—C15—N6	0.05 (13)
C2—N1—C6—C5	−0.35 (16)	C16—N7—C15—N4	−179.50 (11)
Co1—N1—C6—C5	173.82 (8)	N6—N5—C16—N3	178.62 (11)
C2—N1—C6—C7	178.28 (9)	N6—N5—C16—N7	0.70 (12)
Co1—N1—C6—C7	−7.55 (12)	C15—N7—C16—N5	−0.49 (13)
C4—C5—C6—N1	−0.37 (17)	C15—N7—C16—N3	−178.47 (11)
C4—C5—C6—C7	−178.81 (10)		

### Hydrogen-bond geometry (Å, °)

**Table:**

*D*—H···*A*	*D*—H	H···*A*	*D*···*A*	*D*—H···*A*
N3—H1N3···O10^i^	0.841 (19)	2.249 (18)	3.0304 (15)	154.5 (17)
N3—H1N3···N8^i^	0.841 (19)	2.47 (2)	3.1379 (15)	136.5 (16)
N3—H2N3···O11^i^	0.86 (2)	2.28 (2)	2.9563 (15)	135.7 (17)
N4—H1N4···O2	0.85 (2)	2.072 (19)	2.9084 (14)	169 (2)
N4—H2N4···O1W	0.85 (2)	1.96 (2)	2.8057 (16)	172 (2)
N6—H1N6···O3^ii^	0.877 (18)	1.870 (18)	2.7368 (12)	169.7 (16)
N7—H1N7···O7	0.85 (2)	1.89 (2)	2.7245 (13)	167 (2)
O10—H1OA···O7^i^	0.79 (2)	1.86 (2)	2.5775 (13)	152 (3)
O1W—H1W1···O6^iii^	0.78 (2)	2.06 (2)	2.8315 (14)	172 (2)
O1W—H2W1···O1^ii^	0.82 (3)	2.23 (3)	2.8901 (14)	138 (2)
O2W—H2W2···O8^iv^	0.87 (3)	1.67 (2)	2.5279 (13)	170 (2)
O2W—H1W2···O5^v^	0.98 (3)	1.48 (3)	2.4622 (13)	177 (3)
O2W—H3W2···O6^ii^	0.87 (2)	1.71 (2)	2.5746 (13)	174 (2)
O12—H12B···N5^vi^	0.89 (2)	1.76 (2)	2.6303 (14)	169 (2)
C3—H3A···O9^vii^	0.95	2.52	3.1725 (14)	126
C5—H5A···O9^i^	0.95	2.59	3.5380 (15)	175
C10—H10A···O1^viii^	0.95	2.38	3.2669 (14)	156
C19—H19A···O2W	0.95	2.50	3.4269 (15)	165

Symmetry codes: (i) −*x*+2, −*y*+1, −*z*+1; (ii) *x*, *y*−1, *z*; (iii) −*x*+1, −*y*+1, −*z*; (iv) *x*+1, *y*−1, *z*; (v) *x*+1, *y*, *z*; (vi) *x*, *y*+1, *z*; (vii) *x*−1, *y*+1, *z*; (viii) −*x*, −*y*+1, −*z*.
